# Variability of Water Chemistry in Tundra Lakes, Petuniabukta Coast, Central Spitsbergen, Svalbard

**DOI:** 10.1100/2012/596516

**Published:** 2012-05-01

**Authors:** Małgorzata Mazurek, Renata Paluszkiewicz, Grzegorz Rachlewicz, Zbigniew Zwoliński

**Affiliations:** Institute of Geoecology and Geoinformation, Adam Mickiewicz University, Dzięgielowa 27, 61-680 Poznań, Poland

## Abstract

Samples of water from small tundra lakes located on raised marine terraces on the eastern coast of Petuniabukta (Ebbadalen, Central Spitsbergen) were examined to assess the changes in water chemistry that had occurred during the summer seasons of 2001–2003 and 2006. The unique environmental conditions of the study region include the predominance of sedimentary carbonate and sulphate rocks, low precipitation values, and an active permafrost layer with a maximum thickness of 1.2 m. The average specific electric conductivity (EC) values for the three summer seasons in the four lakes ranged from 242 to 398 *μ*S cm^−1^. The highest EC values were observed when the air temperature decreased and an ice cover formed (cryochemical effects). The ion composition was dominated by calcium (50.7 to 86.6%), bicarbonates (39.5 to 86.4%), and sulphate anions. The high concentrations of HCO_3_^−^, SO_4_^2−^, and Ca^2+^ ions were attributed to the composition of the bedrock, which mainly consists of gypsum and anhydrite. The average proportion of marine components in the total load found in the Ebbadalen tundra lake waters was estimated to be 8.1%. Precipitation supplies sulphates (as much as 69–81%) and chlorides (14–36%) of nonsea origin. The chief source of these compounds may be contamination from the town of Longyearbyen. Most ions originate in the crust, the active layer of permafrost, but some are atmospheric in origin and are either transported or generated in biochemical processes. The concentrations of most components tend to increase during the summer months, reaching a maximum during freezing and partially precipitating onto the bottom sediments.

## 1. Introduction

High Arctic lakes, a prevalent and highly sensitive type of polar wetland, are affected by climate change and variations in geomorphic processes [1–3]. Global warming can impact polar water bodies by causing them either to disappear as a result of more intense evaporation from tundra surfaces near glaciers or to proliferate as a result of increasingly frequent and prolonged periods of permafrost thawing in continental areas.

These lakes collect water from various sources, but the majority comes as surface runoff from melting snow cover [4, 5], inflowing streams, and the seasonal thawing of permafrost (suprapermafrost groundwater [5, 6]). The sources of the water inputs for such lakes are reflected in their chemical compositions which, in turn, affects the types of geoecosystems they feature and the biodiversity of their indigenous flora. Lakes change size due to evaporation, enhanced vertical seepage into the thawing substrate, and increased internal drainage from the melting permafrost [7].

The coastal wetlands of Central Spitsbergen include tundra lakes located within the same zone as isostatically elevated seashores. As a result of their transitory location, the lakes are influenced by the littoral and paraglacial geoecosystems and occasionally by the proglacial and glacial geoecosystems. Zwoliński [8, 9] classifies the longer- and shorter-term presence of minerals in the lakes as part of the redeposition cascade that occurs in the sedimentary cycle of the polar oasis. During this cycle, sediments undergo various hydrobiogeochemical transformations in the solutions and diagenesis of muddy bottom sediments. The seasonal nature and related hydrochemical dynamics of water in the tundra lakes play a role in the paraglacial evolution of minerals.

In the classification of the geoecosystems of Ebbadalen (Ebba Valley, Central Spitsbergen), an individual geoecosystem composed of raised marine terraces dotted with tundra lakes was identified [10]. This distinct geoecosystem of tundra lakes plays an essential role in water circulation and in the balance of matter in this region. Because the High Arctic wetlands are difficult to access, there have been few reports on the present state and evolution of tundra lake geoecosystems and few studies containing data that are useful in forecasting future changes in such lakes. Detailed studies of seven shallow tundra lakes on raised marine terraces in Ebbadalen revealed considerable hydrochemical discrepancies [11]. The results indicate how the ionic content of lake waters can change based on the influence of various types of alimentation by snow melt and permafrost degradation. This process is also influenced by bedrock composition and biogenic processes. Environmental studies were conducted to examine twenty-three lakes along a south-north transect on the west coast of Svalbard in the summer season of 1995 [12]. Following this one-time hydrochemical mapping procedure, the lakes were classified and a hydrochemical group of nine lakes on marine terraces was identified. The water in these lakes had relatively high conductivity, alkalinity, Ca^2+^, Mg^2+^ and pH values. The catchments of these lakes are located on the strandflat, which is covered with vascular plants and features a carbonate-rich bedrock.

Following Stankowska's [11] studies, we investigated tundra lake geoecosystems from 2001–2003 and in 2006. Three of the seven lakes studied by Stankowska had dried out completely. This finding is consistent with those of studies conducted in other regions of the Arctic with thawing permafrost, in which bodies of water have disappeared as a result of global warming [2, 3, 13].

The main objectives of this paper are (1) to describe the seasonal chemical variability of water in tundra lakes as a function of various features of the Arctic environment and (2) to determine the ways in which local factors, sources and methods of delivery of dissolved matter, and catchment processes affect lake water chemistry. An understanding of the changes in the chemical composition of freshwater will help make it possible to identify the role that lakes play in the paraglacial evolution of bottom sediments.

## 2. Study Area

**Figure 1:**
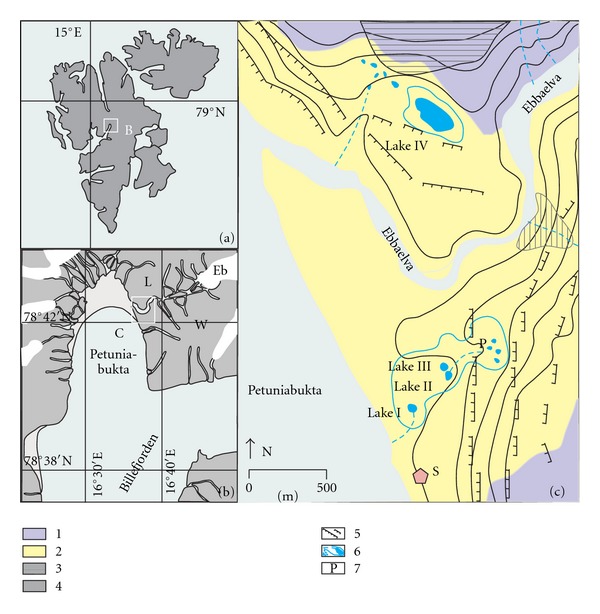
Location of the study area in context of Spitsbergen (a), Billefjorden (b), and Petuniabukta (c) Eb: Ebbabreen; W: Wordiekammen; L: Løvehovden; S: Skottehytta meteorological station. Lakes I–IV: tundra lakes described in this study; 1: slope deposits; 2: marine shore deposits; 3: talus cones; 4: alluvial fans; 5: marine terraces edges; 6: lakes with catchment divides, episodic streams, and lakes; 7: location of piezometers within the wetland.

The study area is located on the northeastern tip of Billefjorden, a branch of Isfjorden, the largest fjord system on the mid-western coast of Spitsbergen (Figure 1). The eastern coast of Petuniabukta, located at the mouth of Ebbadalen, forms a step-like system of raised marine terraces that is characteristic of the post-Pleistocene shoreline of Svalbard [14, 15]. The lowest erosion-accumulation terraces from the mid- to late-Holocene lie at an altitude of 5 to 16  m a.s.l. [16–20]. 

Geologically, the eastern coast of Petuniabukta is dominated by the Ebbadalen Formation, which is composed of Middle and Upper Carboniferous carbonates, anhydrites, gypsums, sandstones and conglomerates [21]. The lower terraces contain sand and gravel sediments originating from local waste materials and, to a lesser extent, from crystalline Heckla Hoek rocks. The higher terraces feature intertwined facies of coastal and slope sediments derived from adjacent outcrops of Ebbadalen Formation sedimentary rocks. According to Gulińska et al. [22], the soils on the raised marine terraces are loose and poorly developed. The sparse vegetation of dry deflational lichen-moss tundra ecosystems mainly includes calcicolous *Dryas octopetala*, *Salix polaris,* and various *Saxifraga* species, all of which are partly interspersed with lichens. Such habitats are predominant in the arid quasicontinental inner region of Spitsbergen [23].

The Ebbadalen tundra lakes occupy shallow depressions and are underlain by mineral soils or thin peat in permafrost terrain [24]. Detailed weekly studies of four of these lakes (Figure 1) were conducted in the years 2001–2003. The lakes are either closed basins or basins with a temporary outflow. Two of them (Table 1), referred to as Lakes II and III, periodically merge into a single body of water (II/III). This was observed in 2003. Lakes I, II, and III are located south of Ebbaelva on raised Holocene marine terraces that cascade down towards the sea from a height of approximately 30  m a.s.l. at the foot of the Wordiekammen massif. Featuring a slight incline, the terraces are covered with a surface network of runoff creeks that connect individual lakes during times of high water and form pronounced erosion cuts in the scarps that separate successive terrace levels. The largest of the lakes, Lake IV, is situated north of Ebbaelva at the foot of the Løvehovden slopes. The terrace of this lake is morphologically diverse due to the presence of outcrops of greatly weathered and crumbling residual erosional bedrock remnants on its surface, which mark the southern and western limits of the lake's catchment. In the north, the adjoining Løvehovden slopes are slightly tilted, creating an area that feeds water and sediments into the lake. Runoff from the slopes of the surrounding massifs results in the deposition of fine mineral layers intercalated with layers of chemical origin (mostly composed of calcite and gypsum flakes) and organic origin (generated mainly by bird guano and plant remnants).

**Figure 2:**
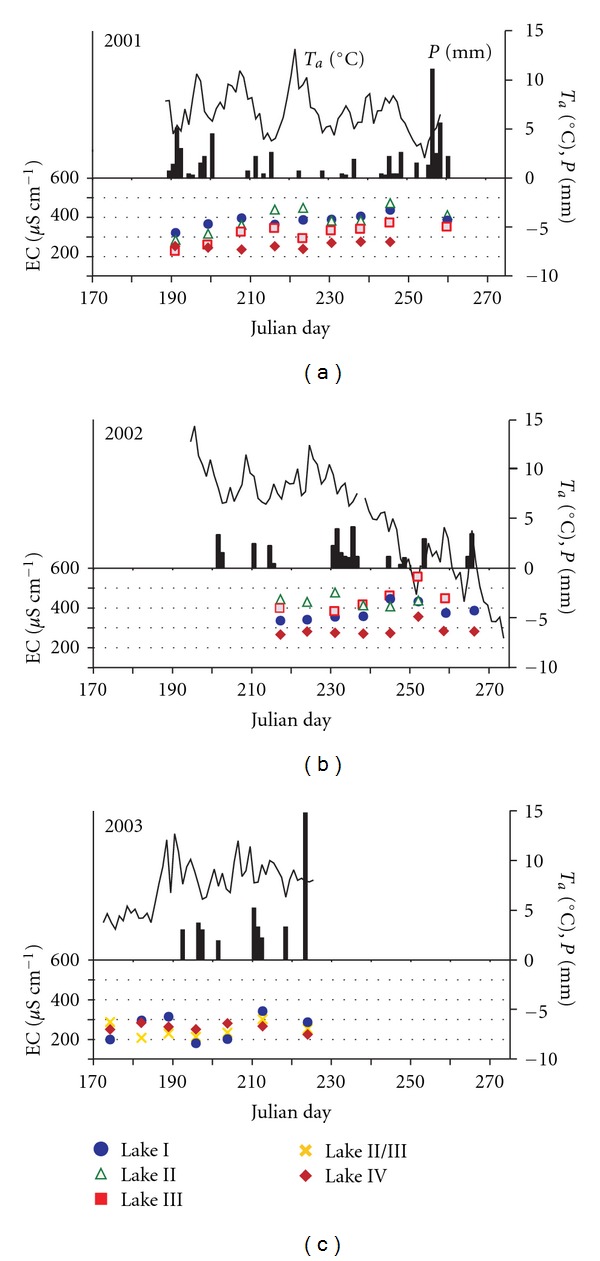
Mean daily air temperature *T*_*a*_ and daily precipitation *P* at Skottehytta and specific electric conductivity EC in Lakes I–IV during summer 2001, 2002, and 2003; reference period from June 19 (170 Julian day) to October 2.

A comparative analysis of the thermal conditions in Skottehytta and Longyearbyen [25] revealed many similarities; however, Skottehytta is warmer in the summer by 1-2°C and colder in the winter by 2–4°C. This discrepancy is attributed to Skottehytta's more continental climate [25, 26]. During the summer period of this study, from 7 July to 17 September 2001, the observations conducted at the Skottehytta meteorological station (Figures 1 and 2) revealed an average daily air temperature of 6.7°C and total precipitation of 59.5 mm. From 6 July to 30 September 2002, the average temperature was 5.3°C and the total precipitation was 34.5 mm. During the summer of 2003 (21 June to 13 August), the average air temperature was 7.6°C and the total precipitation was 46.1 mm. During the corresponding 2006 season, the average daily temperature was 6.7°C and the total precipitation was quite low at 19.4 mm. The variable meteorological conditions were clearly reflected in the fluctuations in the water levels of the lakes [24] and in the humidity of the substrate. Permafrost thaws to a depth of 1.0–1.2  m; this process usually stops at the end of August or the beginning of September [25]. Soil temperatures, even in a 5 cm subsurface layer, reached a maximum of 15°C during the summer seasons for which measurements were taken.

The study region of Central Spitsbergen is characterised by the dry climatic conditions of the High Arctic, with the highest rates of glacier decay and the rapid growth of ice-free areas [26, 27]. In the given subzone on the coast of Petunia Bay, various factors control the development of Arctic wetlands, primary the hydrological regime and water chemistry.

## 3. Field and Laboratory Methods

During several expeditions (2001–2003), studies of the spatial and temporal fluctuations in the chemical composition of water in the four selected water bodies were conducted. Additional hydrochemical mapping was performed on 1st September 2006. During the summer of 2001, weekly measurements of lake water levels were taken. Similarly, daily measurements of water levels and water temperature were taken in Lake I in 2006. Water samples were collected weekly in the morning from the middle of the lakes at half-depth from 7th July to 13th September 2001, from 8th August to 16th September 2002, and from 23rd June to 12th August 2003. On 9th September 2002, samples were taken from below the ice surface of the lakes. The temporal distribution of the water sample, the collection times, and the water parameter measurements show that the observations covered the entire Arctic summer. The temperature, pH, and specific electric conductivity (EC) of the water were measured in the field using a CX-401 multifunctional portable meter with accuracy levels of ±0.1°C, 0.1 pH units, and 0.1%, respectively. The EC values were automatically corrected to a standard value of 25°C.

Water samples were immediately filtered with 0.45 *μ*m membrane filters (Whatman) and stored in polyethylene bottles in the dark at approximately 4°C. For the cation analyses, the samples were acidified with HNO_3_. The bicarbonate concentration of the filtered samples was determined within a day of collection via titration with 0.05 M HCl [28] and a methyl orange indicator. The level of precision error was <7%. More chemical analyses were performed in the laboratory at the Institute of Paleogeography and Geoecology at Adam Mickiewicz University in Poznań. The concentrations of Ca^2+^ and Mg^2+^, which indicated the absorption mode, and the concentrations of Na^+^ and K^+^, which indicated the emission mode, were determined via atomic absorption spectrometry (AAS) using a Varian SpectrAA 20 Plus. The analyses exhibited a precision level of 0.001 mg L^−1^. The concentrations of chlorides and sulphates were analysed via ion chromatography (IC) using a DX-120 Dionex with an anion column, a suppressor mode, and an Na_2_CO_3_/NaHCO_3_ eluent. The uncertainty of the findings, expressed as a relative standard deviation (RSD), did not exceed 10% for the examined anions. The accuracy of the ion concentration measurements was verified against the specific electric conductivity measurements and based on the ion balance [29]. The degree of charge balance error (CBE), which was less than 5%, was considered acceptable. There were 24 samples with a pH greater than 8.3 that did not meet this condition. In such reactions, bicarbonate ions are accompanied by carbonate ions, but the concentration of the latter was not determined. The means and standard deviations of the samples and the number of samples used are listed in Table 2. The saturation index for calcite (SI_C_) was computed using the PHREEQC program [30].

## 4. Results

Water is typically circulated in nonglacierised Arctic catchments from June to September [31]. During the Arctic summer, tundra lakes dwindle, some to the point of vanishing periodically or permanently. The latter distinction depends on their source of alimentation, evaporation levels, and the intensity of the gradual cessation of the water supply from the active layer of permafrost [24]. The source of a lake's water supply is essential in determining its water quality. In 2001, the weekly water levels in Lakes I, II, III, and IV fluctuated by 2-3 cm on average, with a maximum fluctuation of 14 cm. In 2006, daily fluctuations in Lake I did not exceed 2 cm. In July 2006, the water table decreased by 25 cm and then recovered by 10 cm during August. The water temperatures in the lakes ranged from 4 to 17°C in 2001 and from 1.6 to 16.7°C in 2006. The lakes froze slowly during September.

All the investigated tundra lakes showed varying dissolved salt concentrations and ion compositions, as determined from 87 water samples (Table 2). The water was slightly to moderately alkaline, with pH values ranging from 7.62 to 8.73. From 2001 to 2003, the specific electric conductivity values fluctuated between 203 and 559 *μ*S cm^−1^. The average values for the three summer seasons in the four lakes ranged from 242 to 398 *μ*S cm^−1^. For the sake of comparison, Table 2 provides the chemical compositions of shallow lakes found on tundra terraces along the western coast of Spitsbergen [12] and on the terraces of Petunia Bay [11]. The 2006 hydrochemical mapping results for the 4 investigated lakes and 12 other small ponds located on marine terraces along the eastern coast of Petuniabukta reveal significant differences in the proportions of HCO_3_^−^, SO_4_^2−^, and Ca^2+^ ions [24]. Elevated concentrations of these ions are the effect of the limestone-anhydrite bedrock, whereas lower levels of these ions in total dissolved loads indicate the influence of other sources of ions and alternative water supply routes.

Low specific electric conductivity values (between 233 and 355 *μ*S cm^−1^) were observed during the summer seasons in 2001, 2002, and 2003 in Lake IV, the largest of the four lakes, which is located at the foot of the Løvehovden ridge (cf. Figure 1). The lowest EC values (209–268 *μ*S cm^−1^) were recorded in the combined Lakes II/III in 2003. In the previous years, Lakes II and III were separated and their waters showed higher levels of mineralisation. The lowest water level, which rendered Lake III completely dry for a few days, was recorded in 2002.

**Figure 3:**
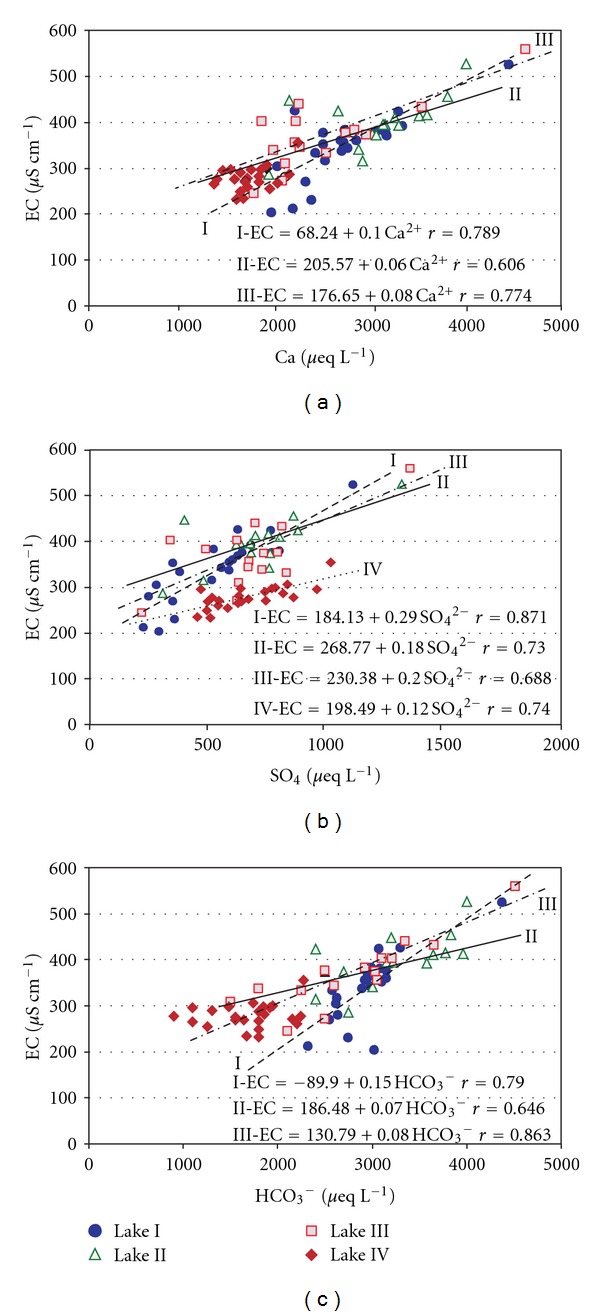
Relationships between specific electric conductivity EC and concentration of selected ions for tundra lakes with regression lines and equations: (a) Ca^2+^, (b) SO_4_^2−^, and (c) HCO_3_^−^.

In the waters stored in Lakes I, II, and III, there was a significant relationship between the specific electric conductivity and the concentrations of bicarbonate and sulphate ions (Figure 3). Lake IV was the only lake that exhibited no significant link between the EC and the HCO_3_^−^ ion concentration. The EC in this lake was a function of the chloride ion content and exhibited a correlation coefficient of 0.66 (*P* ≤ 0.005).

**Figure 4:**
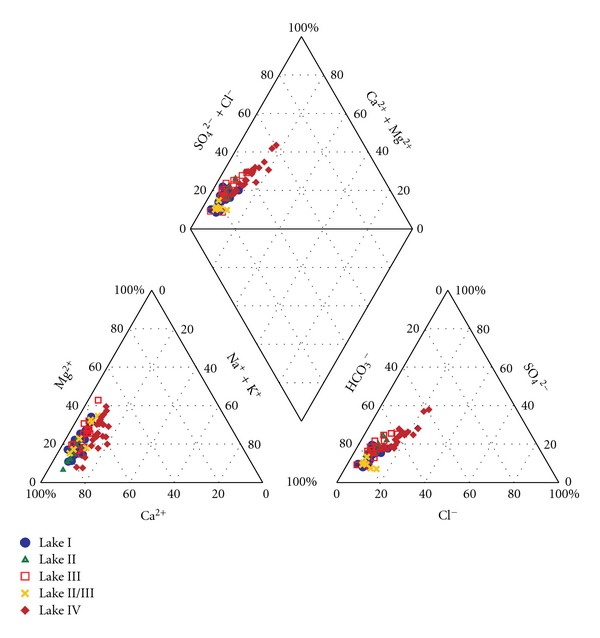
Piper diagrams showing chemical composition of water in tundra lakes I, II, III, II/III, and IV, the vicinity of Petuniabukta.

**Figure 5:**
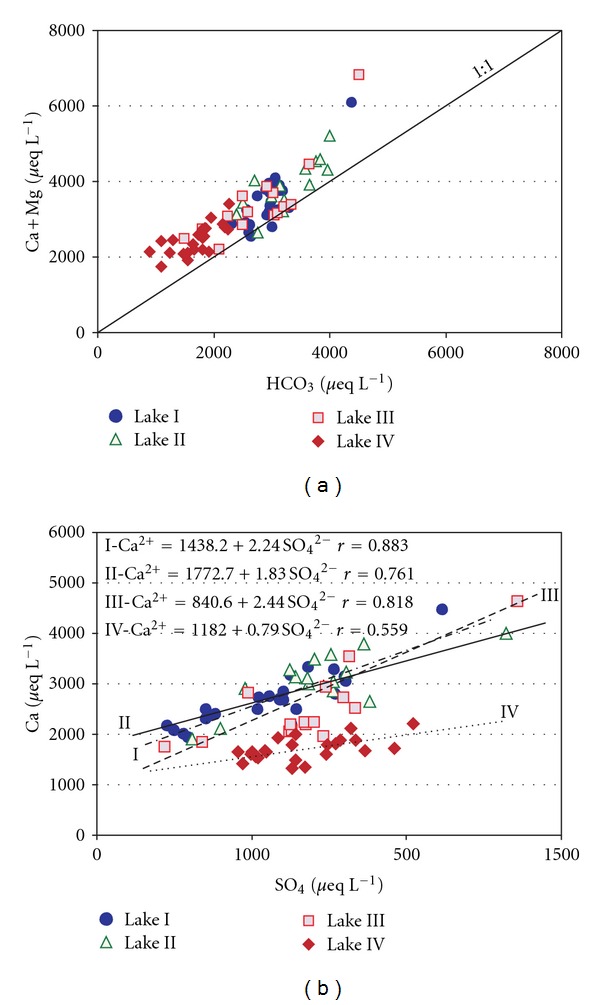
Scatter plots showing relationships between: (a) HCO_3_^−^ – [Ca^2+^ + Mg^2+^] and (b) SO_4_^2−^ – Ca^2+^.

**Figure 6:**
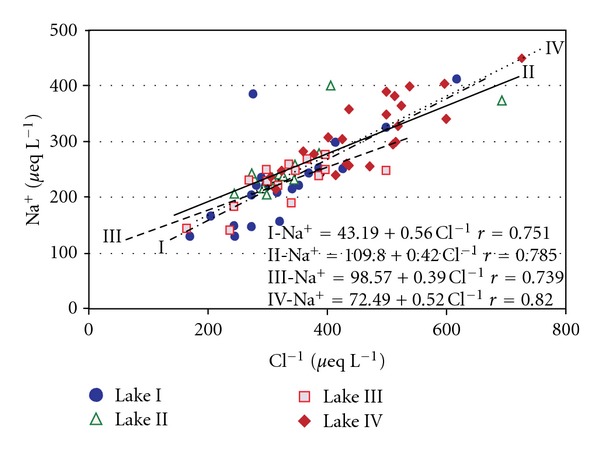
Relationships between concentration of Cl^−^ and Na^+^ with regression lines and equations for tundra lakes.

The waters of these lakes can be classified in the hydrochemical categories of HCO_3_–Ca, HCO_3_–Ca–Mg, and HCO_3_–SO_4_–Ca–Mg. Based on four measurements taken in 2001, the water of Lake IV could be classified as HCO_3_–SO_4_–Cl–Ca–Mg (Figure 4). Regardless of location, the ion content of the lakes was dominated by calcium cations and bicarbonate anions. Bicarbonate ions accounted for 40 to 86% of the total anions, whereas calcium ions accounted for 51 to 87% of the total cations. The (Ca + Mg)/HCO_3_ ratio was greater than one (Figure 5(a)), indicating that the total water hardness in the lakes was caused by the dissolution of calcium carbonate. The difference between the HCO_3_^−^ concentrations and the sum of Ca^2+^ + Mg^2+^ must be balanced primarily by sulphates. The positive relationship between Ca^2+^ and SO_4_^2−^ is apparent in Figure 5(b) and is suggested by correlation coefficients ranging from 0.559 (*P* ≤ 0.005, Lake IV) to 0.883 (*P* ≤ 0.005, Lake I). This trend supports the hypothesis that these chemical elements have the same source. Sulphates are the second most important anion in lake water. They are responsible for 7 to 40% of the total ion content. Fluctuations in the concentration of SO_4_^2−^ also suggest that significant relationships exist between sodium and potassium ions in Lakes II and IV. Strong links between the sodium (Figure 6) and potassium ions (except in Lake II) were indicated by the Cl^−^ ion.

The saturation index for calcite (SI_C_) ranged from −1.14 to 0.93 and was 0.04 on average. A chemical equilibrium, as indicated by values between −0.5 and 0.5, was observed in 52% of the samples. In 20% of the measurements, the index was negative and below −0.5, indicating that the water was unsaturated with respect to calcite. Positive SI_C_ values greater than 0.5 were observed in 28% of the samples, indicating that the water was oversaturated and that mineral deposition was possible, especially in the form of CaCO_3_. The values were mainly positive in 2002, consistent with the peak water mineralisation conditions observed during the monitoring period.

The concentrations of most chemical components slowly increased from the beginning of the ablation season to the peak of the summer season. The highest specific conductivity values were observed when the air temperature decreased in September 2002 (Figure 2), and an ice cover formed. During this period, the conductivity level rose from 295 to 355 *μ*S cm^−1^ in all of the lakes and the corresponding figure for Lake IV increased from 395 to 526 *μ*S cm^−1^. The increase in air temperature led to the melting of the first autumn ice cover and the reestablishment of a lower conductivity (EC) value similar to those observed prior to the formation of the ice cover.

## 5. Discussion

The chemistry of the waters of these lakes may be shaped by different cascades of Ebbadalen geoecosystems [10] and by marine aerosols, atmospheric deposition, chemical weathering, surficial processes, periglacial activity, and biological inputs [24, 32]. Depending on the geological structures and the waste and soil cover on the raised marine terraces and in the surrounding areas, the waters that reach the tundra lakes transport varying proportions of specific chemicals. These proportions are important for the hydrobiogeochemical transformations that occur in Arctic lakes because the dissolved substances in the lakes can modify the properties of the lake sediments, which are used as a basis for analyses of environmental and palaeogeographical changes. There is no information in the literature about the quantitative inputs of marine and atmospheric components into polar lake waters; hence, this study attempted to establish a valuable data set.

### 5.1. Sources of Marine and Atmospheric Ions

All of the investigated lakes are located at elevations of less than 9  m a.s.l. and at distances from the seashore of less than 800  m (Table 1). Therefore, they are affected by marine aerosols and sea spray. Ions of marine origin, such as chloride and sodium ions, are delivered to the coastal wetland lakes directly in aerosols and precipitation and possibly indirectly by surface and subsurface runoff. In a significant majority of the water samples, the Cl^−^ to Na^+^ ratio exceeded the widespread sea water level of 1.14. This finding is consistent with Krawczyk et al.'s [33] suggestion that chlorides can originate from sources other than sea salt. The average proportion of chlorides derived from marine aerosols in the lake waters was estimated to be approximately 80%. Based on the ionic equivalent ratios of Ca^2+^, Mg^2+^, K^+^, and SO_4_^2−^ to chlorides of sea origin [34, 35], the concentrations of calcium ions of sea origin were estimated to be approximately 0.5%, 0.9%, 18.1%, and 5.3% of the total concentrations of Ca^2+^, Mg^2+^, K^+^, and sulphates, respectively. Analysing the sources of the loads transported by the river Bayelva (NW Spitsbergen) during the autumn season in 2000, Krawczyk et al. [33] estimated that 3.2 or 3.4% of the total solute load was of marine origin depending on the calculation method. The average share of the marine components in the total amount of ions in the Ebbadalen tundra lake waters was higher at 8.1% or 18 mg L^−1^. In addition to sulphates and chlorides, the components of atmospheric origin also included some bicarbonate ions, which are products of abiotically and biotically mediated soluble atmospheric CO_2_.

Precipitation in central Spitsbergen is influenced by sea aerosols, pollutants transported by air over long distances and local contamination from human sources. An analysis of the chemical composition of the precipitation collected at the Skottehytta station and at other monitoring sites in Spitsbergen [36–38] showed that in addition to contributing ions of marine origin, precipitation contributed human-generated sulphates and chlorides to the lakes (Table 3). The average concentration of chlorides was 4.96 mg L^−1^, and the nonsea annual loads ranged from 14 to 36%. The sulphates that did not originate from sea salt accounted for up to 81% of the total sulphates in precipitation that derived from human sources.

On the eastern coast of Petuniabukta, the total loads of salt transported via precipitation were approximately 848 kg km^−2^, 500 kg km^−2^, and 1,017 kg km^−2^ during the summer seasons of 2001, 2002, and 2003, respectively, with crustal Na^+^ and K^+^ ions and nonmarine (i.e., anthropogenic) Cl^−^ and SO_4_^2−^ ions also contributing to the total solute load. Studies of water chemistry in Hornsund have suggested that some of the sulphates that did not derive from sea salt had anthropogenic origins. Depending on weather conditions, they may have come from anthropogenic air pollution from distant sources [36], mainly Siberia and the Kola Peninsula in northeastern Europe. Atmospheric deposition in sediments in lakes along the western coast of Spitsbergen [12] presumably occurred through a combination of local sources, including domestic coal mining activities and settlements and long-range transport. The cessation of coal mining in Pyramiden in the spring of 1998 greatly reduced the impact of local sources in the Petunia Bay area. The surveyed area, however, continues to be affected by contamination transport from the Isfjord coasts, mainly that of Longyearbyen, within a radius of 60 to 80 km [39].

The supply of elements from precipitation had the most severe impact on the chemistry of water in Lake IV, as evidenced by its low specific conductivity, the higher concentrations of Na^+^ and Cl^−^, a correlation between the specific conductivity and the concentration of chlorides, and the lack of such correlations for calcium, magnesium, and bicarbonate ions (Figure 2). These results are affected by the small contribution of underground and surface nourishment from the lake's catchment and the limited supply of crustal ions, as indicated by the level of atmospheric ions in the lake water.

### 5.2. Sources of Crustal Ions

The chemical composition of the water in the tundra lakes and the lakes' high rates of mineralisation, in conjunction with the low ion concentration and the low rates of mineralisation of atmospheric precipitation (approximately 17.5 mg L^−1^) in Svalbard (which is also on the eastern coast of Petuniabukta—cf. Table 3), suggest the existence of significant levels of substances with nonsea and nonatmospheric origins in these lakes. An increased rate of lake water mineralisation during the ablation season (cf. Figure 2) could indicate the influence of evaporation on freshwater chemical composition changes.

Among the anions, bicarbonates were dominant. Their presence, except in the case of those of atmospheric origin, results from the carbonation of carbonates, aluminosilicates, and silicates [33, 37]. These chemical reactions release calcium ions, which are a product of the weathering of carbonate rocks, aluminosilicates and other silicates, anhydrites and gypsum debris in marine terrace deposits, waste cover, and bedrock. Given the contribution of the anthropogenic components carried by precipitation, evaporates are also a source of nonsea SO_4_^2−^ and are balanced by crustal Ca^2+^ cations. In this particular case, sulphates originate from the base of the active layer of permafrost, where anhydrite and gypsum particles of the Ebbadalen Formation contained in the marine terrace material undergo dissolution. This is indicated by the elevated Ca^2+^ and SO_4_^2−^ levels and by representation of the HCO_3_–SO_4_–Ca–Mg hydrochemical group. Additional sources of calcium cations include cryochemical calcite [40, 41], which is redeposited by water and wind from the post-glacial zone of the Ebba glacier. The high pH, EC, and concentration values of the major cations (Ca > Mg > Na > K) and anions (HCO_3_ > SO_4_ > Cl) reflect the high levels of CaCO_3_ in the terrace sediments. Ponds fed by water from calcareous deposits on islands in the Canadian High Arctic were found to have a similar chemical composition [42, 43]. One crustal source of Mg^2+^, Na^+^, and K^+^ ions could be the weathered debris of aluminosilicates and silicates in the metamorphic rocks from the Heckla Hoek Formation.

The Svalbard lakes are potentially sensitive to acid deposition because they are located on deep permafrost and have little exposure to groundwater [44, 45], as in the northern parts of Svalbard [46]. Conversely, alkaline lakes on carbonate bedrock appear to have undergone pH increases in recent times. The concentrations of base cations and slightly alkaline reactions measured clearly indicate the insensitivity to acidification of the lakes located on the marine terraces of Petunia Bay. This finding is also corroborated by the high average ANC (acid neutralising capacity) value found, which reached a maximum of 2.44 meq L^−1^ in the surveyed lakes.

Crustally derived ions are released from permafrost and carried to the lakes in the active layer by suprapermafrost groundwater. A significant pool of solutes has been identified in the near-surface permafrost; these solutes will be released into the active layer during periods of deeper thaw [47, 48]. Thus, in August 2007, the chemical composition of the water from the active permafrost layer was examined at a depth of approximately 1 m in six piezometers near Lake II/III (Figure 1). The water exhibited high mineralisation levels (450–650 mg L^−1^) and substantial differences in the concentrations of the principal ions [49], including cations of calcium, which ranged from 9 to 106 mg L^−1^ (87.7 mg L^−1^ on average), and magnesium, which ranged from 22 to 30 mg L^−1^ (25.2 mg L^−1^ on average). The water also exhibited substantial differences in the concentrations of the anions of bicarbonates, which ranged from 256 to 366 mg L^−1^ (313.1 mg L^−1^ on average), and sulphate, which ranged from 21 and 73 mg L^−1^ (44.2 mg L^−1^ on average). The high concentrations of these ions in permafrost water indicate that suprapermafrost transport plays a substantial role in the alimentation of the tundra lakes. These concentrations also show that this water has a high dissolving capacity combined with readily accessible soluble compounds in the substratum deposits. Depending on the geological structure and the diverse weathering processes in the areas surrounding the raised marine terraces [26], the solutions that flow into the tundra lakes in suprapermafrost water may have varying levels of crustal components.

### 5.3. Sources of Biogenic Ions

Saturated areas around the Ebbaelva lakes often feature zones of richer tundra vegetation including various species of lichens, liverworts, moss, grass, and herbs, all of which are a source of components of biogenic origin. In surficial lacustrine deposits, the organic matter content can reach 23% [24]. Lim et al. [43] found that smaller ponds with vegetated catchments have more dissolved organic matter. A factor that contributes to the eutrophication of soil and accelerated plant growth is the presence of birds and their guano around the surveyed lakes of Petuniabukta. Despite the slow rate of eutrophication, the decomposition of organic matter in the tundra ecosystem releases biogenic compounds, including phosphates, which were not analysed in this study, and nitrates from the waters that feed the lakes, especially at the end of the polar summer. In Lakes II and III, a mean nitrate concentration of 0.38 mg L^−1^ and a range of 0–1.78 mg L^−1^ were observed in 2003. These results are supported by research on nonglacierised catchments in Petuniabukta and various parts of Spitsbergen [32, 50, 51], which show that the quantity of available ions can be modified by the presence of bird colonies that supply such biogenic substances, including nitrates, phosphates, and uric acid.

### 5.4. Changes in Water Chemistry during the Ablation Period

The year-to-year and seasonal changes in the amounts of snowfall and summer rainfall affect the quantities of the surface water and groundwater that reach the tundra lakes, the size and depth of the lakes, and the hydrochemical properties of the lake waters and bottom sediments. The water supply to the tundra lakes is a function of the weather. The weather conditions determine the rate of evaporation, mainly in the ablation season, and the presence of the active layer. In the dry and semidry regions of Central Spitsbergen, the rate of evaporation is relatively high. For instance, the Petuniabukta area was characterised by an evaporation rate of 1.5–1.8 mm per day during the study years (Dragon and Marciniak, personal communication). Smol and Douglas [2] stated that elevated evaporation and low precipitation cause the Arctic lakes to dry up during the polar summer. This phenomenon is also observed in the vicinity of Petuniabukta. High evaporation rates from lake surfaces are also the result of water temperatures that can exceed 10°C in July-August [24]. As the air and water temperatures increase, the water table in the lakes drops, contributing to the gradual saturation of the lake-water components (positive SI_C_ values). This type of saturation was observed until the midsummer of 2002 (5 August 2002), a period that featured low total precipitation and significantly higher temperatures than were recorded in the preceding years (cf. Figure 2).

**Figure 7:**
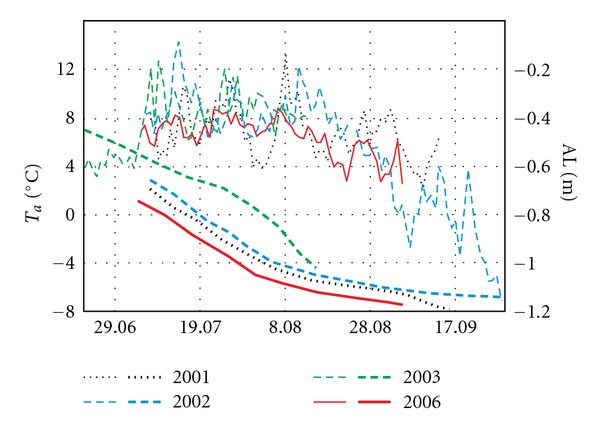
Seasonal thawing of permafrost near Skottehytta on the eastern coast of Petuniabukta, Central Spitsbergen (after [27] and own observations); Ta: air temperature (thin lines), AL: thawing depth (bold lines).

As previously mentioned, weather conditions also control the dynamics of permafrost melting (Figure 7), which overlaps with the evaporation relationships in the studied lakes. In the three observation periods, the 2003 ablation season started on 23 June, a period that featured low temperatures and a relatively thin active layer of 45 cm (compared with the maximum thickness of 102 cm on 13 August 2003). The parameters of the water, particularly conductivity, were generally low, possibly because the late snowmelt provided more meltwater and diluted the wetland water in midsummer. Similarly, low EC values were recorded in 2001 in the initial measurement period that began on 9 July. The thickness of the active layer was 69 cm at that time, and it increased during the summer to reach a maximum of 120 cm on 30 August (Figure 7).

The concentrations of ionic components in lake waters increase during the polar summer towards the end of the ablation period, when the depth of the active layer reaches its maximum. Hodson et al. [37] theorised that factors such as extended rock-water contact times and high soil-water *p*(CO_2_) could enhance chemical weathering within the active layer. As the permafrost table continues to degrade, the specific conductivity of the lake water continues to increase, as do the amounts of calcium, magnesium, bicarbonates, and sulphates (cf. Figure 2). The properties of lake water indicate that permafrost plays an important role in adding soluble salts to the active layer ([52], cf. [1]). The amount and nature of the migrating ions depend on the geochemistry of the underlying permafrost. In the studied wetland lakes, the substrate is dominated by readily soluble carbonate and sulphate rocks from the Ebbadalen Formation. Whenever a lake loses all of its water, a chemogenic veneer is deposited on its bottom. As water evaporates from the emerging bottom, calcium carbonate and sulphates precipitate as white flakes. SI_C_ index values that are greater than 0.5 reveal oversaturation with calcium carbonate, which can lead to its precipitation. Calcium-sulphate deposits are a highly soluble precipitation product and can influence water chemistry by redissolving under favourable conditions. These conditions include rain events that activate infiltration or leaching by surface water, especially at the beginning of the ablation season, when surface runoff dominates in the lake supply [49]. If the temperature falls below 0°C and causes the lake to freeze, the concentration of salt in the remaining bottom water strata increases due to cryochemical processes [9, 41, 53].

## 6. Conclusions

The results of the hydrochemical studies conducted in Spitsbergen expand the range of limnological data sets from the High Arctic and reveal more variation than did previous reports. The major ion concentration, pH, and EC values for the High Arctic tundra lakes studied are based on observations from several years, which is rare in Arctic regions, where limnological studies are usually based on single observations (compare [54]). The temporal variability of the chemical composition of the analysed waters reflects variations in the operation of the geoecosystems during the polar summer and suggests that caution should be exercised when interpreting single mappings of High Arctic lakes.

The tundra lake geoecosystems found on raised marine terraces exist from June to August and occasionally into September. Thus, our data accurately reflect polar summer conditions. The water level in the tundra lakes usually fluctuates seasonally or annually with atmospheric precipitation, evaporation, snow cover and permafrost melting, and seepage losses. The major ion concentrations and conductivities found in the Petuniabukta lakes were relatively high compared to those of the lakes on the tundra terraces along the western coast of Spitsbergen, as examined by Birks et al. [12]. The differences in the proportions of HCO_3_^−^, SO_4_^2−^, and Ca^2+^ ions reflect the lithology of the bedrock, in which limestone and dolomite are accompanied by gypsum and anhydrite in waste cover. The chemistry of lake water is affected by a combination of marine (8.1%), atmospheric, crustal/denudational, and biological factors.

The large values for the major ions pH and EC indicate that alimentation occurs mainly from the active layer of permafrost, whereas the chemical composition of the lake water reflects that alimentation occurs through the underlying bedrock and geochemical transformations that occur during weathering processes. The chemical composition of the water is a consequence of dilution with poorly mineralised water during the thawing of snow cover, the leaching of salts from the active layer of permafrost, and salt enrichment caused by evaporation and matter concentration during freezing. Because of the low total volume of summer rainfall, precipitation in the High Arctic wetlands plays a relatively limited role. An analysis of the chemical composition of the water revealed that the dissolved compounds were mainly delivered to the lakes in two ways:

- in water with a long circulation cycle, mostly through suprapermafrost groundwater and subsurface waters that transport high concentrations of components from the active layer of permafrost and rock waste during periods of permafrost degradation,
- in water with a short circulation cycle in the catchment, mostly from precipitation and surface runoff (overland flow) in waterlogged areas, which may release salts originating from sea aerosols, chemogenic veneer, and biogenic substances from the topsoil in catchments with richer tundra vegetation and man-made contaminants.

The climatic changes observed in the Arctic during the last decades mainly affect the former pathway of water delivery to the tundra lakes. The warming observed in the northernmost latitudes accelerates the thawing of permafrost and evaporation in wetlands [3, 13, 55]. Thus, hydrochemical changes in tundra lakes are caused by local, regional, and global changes in the environment.

## Figures and Tables

**Table 1:** Lake basins and catchment characteristics of tundra lakes.

Name	Latitude	Longitude	Altitude	Surface catchment area	Water area^a^	Max depth	Distance from sea	Outflow	Percent of vegetation
	deg N	deg E	m a.s.l.	m^2^		m		—	%
Lake I	78°42.11′	16°36.60′	4	13690	1 112	0.51	80	Episodic in- and outflow	90
Lake II	78°42.16′	16°36.95′	5	9540^b^	596	0.22	160	Episodic outflow	90
Lake III	78°42.18′	16°36.88′	5		386	0.13	160	Episodic in- and outflow	90
Lake IV	78°42.58′	16°37.04′	9	34126	5579	0.53	800	Isolated	60

^a^Status as measured on July 9, 2001.

^b^High water level in lakes II and III leads to the connection of lakes forming one lake {II/III}.

**Table 2:** Statistics for major ions, pH, and specific electric conductivity (EC) data for tundra lakes I–IV for the period 2001 to 2003 and separately for the years 2001, 2002, and 2003; also provided for comparison are the chemical properties of water in Lakes I, III, and IV on 1 September 2006 and (a) in the lakes on the strandflat of Spitsbergen between 28th July and 14th August 1995 [12] as well as (b) in the lakes on terraces around Petunia Bay from 28th June to 27th July 1987 [11].

Lake	Parameter	pH	EC	Ca^2+^	Mg^2+^	Na^+^	K^+^	HCO_3_^−^	Cl^−^	SO_4_^2−^
		—	*μ*S cm^−1^	*μ*eq L^−1^						
Lakes I–IV										

I	Min	7.9	203	1 600	250	129	14	2 320	170	227
I	Mean	8.2	345	2 584	813	230	26	2 934	335	556
I	Max	8.6	524	4 456	1 850	412	37	4 580	618	1 118
I	Mean 2001, *N* = 9	8.0	372	2 819	630	288	30	2 854	406	669
I	Mean 2002, *N* = 8	8.3	387	2 732	985	201	22	3 281	314	648
I	Mean 2003, *N* = 7	8.2	261	2 114	853	190	25	2 640	270	307
I	01/09/2006	—	414	3 340	810	210	30	3 020	490	1 000

II	Min	7.8	286	1 900	220	204	20	2 400	104	192
II	Mean	8.2	398	3 019	823	255	26	3 214	334	700
II	Max	8.6	526	3 992	1 570	401	34	4 000	692	1 322
II	Mean 2001, *N* = 9	8.0	374	2 760	685	251	25	2 861	288	613
II	Mean 2002, *N* = 6	8.3	433	3 409	1 030	261	26	3 743	402	831

III	Min	7.6	244	1 400	60	140	14	1 500	165	219
III	Mean	8.1	371	2 408	853	226	24	2 909	332	673
III	Max	8.5	559	4 632	2 180	276	34	4 520	527	1 359
III	Mean 2001, *N* = 9	7.8	327	2 167	653	234	26	2 442	344	640
III	Mean 2002, *N* = 6	8.2	436	2 770	1 154	215	20	3 608	315	723
III	01/09/2006	—	469	3 670	690	180	50	3 290	430	1 330

II/III	Min	7.8	209	1 560	470	136	12	1 840	195	169
II/III	Mean, *N* = 7	8.3	242	2 057	729	194	24	2 433	259	294
II/III	Max	8.6	268	2 660	1 000	304	32	3 140	410	483

IV	Min	7.7	233	1 320	140	197	28	900	305	457
IV	Mean	8.3	279	1 744	763	314	50	1 692	479	669
IV	Max	8.7	355	3 220	1 200	449	66	2 270	727	1 020
IV	Mean 2001, *N* = 8	7.8	275	1 630	455	352	52	1 319	518	730
IV	Mean 2002, *N* = 8	8.5	303	1 968	920	321	50	1 859	522	747
IV	Mean 2003, *N* = 7	8.3	256	1 620	935	262	49	1 928	384	511
IV	01/09/2006	—	376	2 270	1 100	420	70	2 010	760	1 100

(a) West coast of Spitsbergen										

A	Veslekuplen	8.2	169	853	411	278	13	992	342	42
B	Hamnetangen	8.4	189	863	466	357	15	832	227	42
Q	Ytertjorna	7.9	153	649	348	204	5	664	190	74
R	Spalen	7.7	120	853	183	191	3	432	228	144
S	Vassauga	8.2	243	1 652	900	518	18	1 311	656	308
U	Tenndammen	7.1	323	339	563	2 127	41	220	2 342	327

(b) Petuniabukta										

1	Mean 1987, *N* = 5	8.0	270	1 760	796	704	—	2 254	330	784
2	Mean 1987, *N* = 3	8.4	340	2 513	880	403	—	2 917	270	707
3	Mean 1987, *N* = 3	8.4	383	3 093	807	563	—	3 783	280	400
4	Mean 1987, *N* = 5	8.0	550	4 396	1 112	612	—	3 340	284	2 776
5	Mean 1987, *N* = 5	8.0	558	4 532	1 228	646	—	3 310	272	3 240
6	Mean 1987, *N* = 5	8.0	564	4 636	1 096	534	—	2 610	244	3 352
7	Mean 1987, *N* = 5	7.9	580	5 020	1 068	498	—	2 560	214	3 924

N: number of samples analysed.

**Table 3:** Mean chemical characteristics of rain^a,b^ and snow^c,d^ in Svalbard.

Location	Period	N	Na^+^	K^+^	SO_4_^2−^	Cl^−^	EC
			*μ*eq L^−1^				*μ*S cm^−1^
Skottehytta^a^							

	2001	7	36 (1)	7 (6)	21 (17)	68 (27)	20.3
	2002	9	64 (2)	9 (8)	41 (34)	81 (5)	20.7
	2003	10	99 (3)	8 (6)	37 (26)	141 (29)	31.5

Hornsund^b^							

	2000	18	117 (15)	4 (1)	21 (9)	134 (15)	20.5
	2001	12	40	2	11	44	

Austre Brogger^c^							

	1992	47	90 (0)	4.5 (3)	18 (7)	110 (6)	

Finsterwalder^d^							

	1994 and 1995	41	61 (0)	2.9 (2)	15 (8)	73 (2)	

In parentheses estimated crustal concentrations for cations and nonsea salt concentration for anions are given, N: number of samples analysed.

^a^This paper.

^b^Głowacki and Krawczyk [36], concentrations volume weighted.

^c^Hodson et al. [37].

^d^Wadham et al. [38].
